# Supplementary material for: Prevalence of and factors associated with lipid screening in young people aged 16– to 21 years in the United States: analysis of nationwide cross-sectional data
Source: Lipids Health Dis. 2024 Oct 26;23:350. doi: 10.1186/s12944-024-02336-1 (PMC11514873; doi:10.1186/s12944-024-02336-1)
Supplement: Supplementary file 2 — Supplementary Material 2 [file 12944_2024_2336_MOESM2_ESM.pdf]

Prevalence of and factors associated with lipid...

By: Nianyan Li

As of: Oct 18, 2024 7:56:11 AM

3,737 words - 8 matches - 1 source

Similarity Index
2%

Mode: Similarity Report

paper text:

Prevalence of and factors associated with lipid screening in young people aged 16– to 21 years in the United States: Analysis of nationwide cross-sectional data Abstract Background & Aims: There is no consensus on whether it is worthwhile for young people to undergo lipid screening. An estimate of the national prevalence of lipid screening in this population can serve as a basic parameter in analyses of the utilization and cost-effectiveness of health care services. Previous studies were mostly based on electronic health records or insurance claims data or were restricted to adolescents at younger ages. This study aimed

to investigate the prevalence of lipid screening and 1

the factors associated with it in young people aged 16– to 21 years in the United States based on a nationally representative sample. Methods: A cross-sectional study of 16- to 21-year-old participants from the National Health and Nutrition Examination Survey (NHANES), 2013–March 2020 (four cycles), was conducted.

The prevalence of lipid screening uptake **was** 1

calculated for each cycle as well as for all four cycles combined, and the associations of lipid screening uptake with the covariates were analysed by logistic regression. An appropriate sample weight was incorporated into the analysis as recommended. Results: A total of 3,600 participants were included in the analysis, and 32.02% of them had ever received lipid screening at the time of the survey, with no statistically significant secular changes observed over the four cycles. Among the participants, 28.83% received lipid screening within the past 5 years. In multivariable regression analysis adjusted for all covariates, non-Hispanic black ethnicity (adjusted odds ratio [AOR]: 1.43), having health insurance (AOR: 1.54), obesity (AOR: 1.65) and sufficient physical activity (AOR: 1.36) were significantly associated with a greater chance of ever receiving lipid screening. Conclusions: Nearly one-third of young people aged 16– to 21 years in the United States have received lipid screening. Ethnicity, health insurance, weight problems, and physical activity level were associated with the likelihood of lipid screening uptake. These findings could help people understand the current situation of lipid screening in young people in the United States and may serve as fundamental parameters in the assessment of the utilization and cost-effectiveness of related health care services. I. Introduction Background Dyslipidaemia in children and adolescents is associated with premature atherosclerosis, cardiovascular disease and death in adulthood [1, 2]. Familial hypercholesterolemia and multifactorial dyslipidaemia

are the two main types of dyslipidaemia in this population. According to the evidence reported by the United States Preventive Services Task Force (USPSTF), in children and adolescents in the United States (US), the prevalence of familial hypercholesterolemia ranges from 0.2– 0.4%, and that of multifactorial dyslipidaemia ranges from 7.1–9.4% [3]. Screening is widely implemented to detect and treat diseases early, with the ultimate goal of preventing severe outcomes such as mortality. However, several randomized controlled trials conducted in adults have shown that many screening programs are ineffective in this regard [4-7], and no randomized trials have evaluated the effects of lipid screening on long-term outcomes in children and young people. There are different recommendations for lipid screening

in children and adolescents. The National Heart, Lung, and Blood Institute (NHLBI) 1
recommends universal lipid screening once in children aged 9– to 11 years and

again when they are 17– to 21 years old to identify those with elevated lipid levels, especially those with familial hypercholesterolemia. Selective lipid screening

is recommended for children aged 2 to 8 years and those aged 1

12 to 16 years if they have risk factors such as a family history of cardiovascular diseases or dyslipidaemia, diabetes, hypertension, a body mass index (BMI) ≥85th percentile, and smoking cigarettes [1]. The American Academy of Pediatrics also recommends a selective screening strategy [8]. In contrast, the USPSTF guidelines [2, 9, 10] have consistently suggested that there is insufficient evidence for universal lipid screening in asymptomatic children and adolescents aged ≤20 years and that whether to order a lipid screening test for young patients is at the discretion of clinicians [2]. Given the discrepancy of recommendations by different entities, it is of interest to know the actual rate of lipid screening in children and young people. This information can serve as a basic parameter in analyses of the utilization and cost-effectiveness of health care services. 3/1615 The screening rates reported by previous studies varied considerably from 2–46.8% [11-18]. Older age, overweight or obesity, nonwhite ethnicity, and the presence of some comorbidities have been reported to be associated with higher screening rates [3, 11-13]. However, most of the previous studies were based on electronic health records or insurance claims data from one or several states [13-17]. Furthermore, some studies included only well-child visit records [12, 14, 16, 18] or focused on 9- to 11-year-old children [11, 14-16]. These issues undermined their representativeness of the national population of children and young people, particularly the older children within this population, among whom the prevalence rates of overweight/ obesity and dyslipidaemia are higher [19-21]. Therefore, this study was conducted to evaluate the nationwide prevalence of lipid screening among 16- to 21-year-old young people in the US. As a secondary objective, the factors associated with the uptake of lipids in this population were also explored. The novelty of this study, compared with previous studies, lies in the age range of the study participants, which was less examined, and the national representativeness of the participants, which was rarely achieved. II. Methods Study design and population This was a cross-sectional study based on data from the National Health and Nutrition Examination Survey (NHANES). The

NHANES is a national survey that collects data every 2 years. A complex, multistage and probability sampling strategy is adopted to make the data representative of noninstitutionalized US civilians. The data can be downloaded directly from the NHANES website for free. The sample for the present study was selected from those who participated in any of the four NHANES cycles conducted from 2013–March 2020 before the pandemic. All participants between 16 and 21 years of age were eligible to be included in the study population. Because the questions about lipid screening (“BPQ080” and “BPQ060”) were only asked of those who were aged 16 years or above since 2013, those under 16 years old were not considered in this study. The oldest age recommended for lipid screening in the current guidelines is 21 years [1, 2, 8], and the vast majority of previous studies did not include participants above 21 years of age; therefore, the upper age limit was set at 21 years in this study. The participants who did not answer the two questions were also excluded. NHANES was approved by the National Center for Health Statistics ethics review board, and written informed consent was obtained from all participants or their parents or guardians. This study was approved by the Survey and Behavioural Research Ethics Committee of The Chinese University of Hong Kong (reference no. SBRE-23-0967). Measurements of lipid screening uptake The questions about lipid screening history in the NHANES interviews included “Have you ever been told by a doctor that your blood cholesterol level was high?” (question BPQ080) and “Have you ever had your blood cholesterol checked?” (question BPQ060). Those who answered “Yes” to question BPQ080 were not asked 5/1615 question BPQ060. The participants who answered “Yes” to either of the two questions were considered “ever received lipid screening”. For these people, their responses to “How long has it been since you last had your blood cholesterol checked?” (question BPQ070) were also analysed and reported in this study. Measurements of covariates The following factors were collected from the NHANES and considered potentially associated with the uptake of lipid screening: age, sex, race, physical activity level, health insurance status, diagnosed hypertension, diagnosed diabetes, BMI category, and lipid profile (including total cholesterol [TC], high-density lipoprotein cholesterol [HDL-C], and non-HDL-C). Among them, BMI and lipid profiles were collected at the Mobile Examination Center, whereas the other data were self-reported by participants or their parents or guardians during the questionnaire survey. There were four BMI categories, i.e., underweight, normal weight, overweight, and obese. The cut-off points used for defining the four categories differ between those ≥20 and <20, according to the World Health Organization and the US Centers for Disease Control and Prevention [22, 23]. For those ≥20, the definitions were as follows: BMI<18.5 (unit: kg/m2), underweight; 18.5≤BMI<25, normal weight; 25≤BMI<30, overweight; BMI≥30, obese. For those under 20, the BMI categories were age- and sex-specific and were expressed as percentiles: <5th percentile, underweight; 5th to 85th percentile, normal weight; 85th to 95th percentile, overweight; and ≥95th percentile, obese. For those under 20 years, the BMI categories they fell into could be obtained directly from the response to the question BMDBMIC in the 6/1615 NHANES, whereas for those ≥20 years, the original values of BMI were provided by the NHANES dataset and were divided into different categories by the authors of this study according to the abovementioned definitions. According to the recommendations of the Physical Activity Guidelines for Americans and the World Health Organization [24, 25], children and adolescents aged 5–17 years should perform at least 60 minutes of moderate-to-vigorous physical activities per day, whereas adults aged 18–64 years should perform at least 150 minutes of moderate-intensity or 75 minutes of vigorous-intensity or an equivalent combination of moderate- and vigorous-intensity physical activities per week. For those under 18 years of age, physical activities were categorized into “sufficient” (7 days of ≥60 minutes of moderate-to-vigorous physical activities during the past week) or “insufficient” (less than 7 days of ≥60 minutes of

moderate-to-vigorous physical activities during the past week) directly based on their reporting. For those aged 18 years or above, first, the suggested metabolic equivalent (MET) scores from the NHANES were used to calculate their weekly physical activity level, which was equal to the “suggested MET × weekly frequency × minutes of each physical activity” [26, 27]. The physical activity level was then categorized into “sufficient” (≥600 MET- minutes/week) or “insufficient” according to the World Health Organization standards [25]. The data on low-density lipoprotein cholesterol and triglycerides were available for less than half of the study population in the NHANES, whereas those on TC and HDL-C were available for approximately 90% of the study population. Therefore, 7/1615 only TC, HDL-C, and non-HDL-C (defined by TC minus HDL-C) were included as covariates in this study. For those aged 19 years or younger, according to the National Cholesterol Education Program Expert Panel on Cholesterol Levels in Children and NHLBI report [1, 28], HDL-C >40 mg/dL was considered “normal” and ≤40 mg/dL was considered “low”; TC <200 mg/dL was considered “normal” and ≥200 mg/dL was considered “high”; and

non-HDL-C <145 mg/dL was considered “normal” and ≥145 mg/dL 1

was considered “high”. For those aged 20 years or above, according to the NHLBI report [1], TC <225 mg/dL was considered “normal” and ≥225 mg/dL was considered “high”; non-HDL-C <190 mg/dL was considered “normal” and ≥190 mg/dL was considered “high”; and the cut-off point for HDL-C was the same as that for those ≤19 years of age. A family history of diseases, education level, smoking status, and drinking status may also be associated with the use of lipid screening. However, in the NHANES, questions about the family history of young people focused on diabetes only and did not include dyslipidaemia or cardiovascular diseases. The questions about education level were only asked to those aged 20 years or above. The data concerning young people’s smoking and drinking status can only be accessed on site through the Research Data Center of the National Center for Health Statistics. Therefore, these covariates were not included in this study. Data analysis For each variable, the frequencies and percentages are presented. The prevalence of lipid screening was calculated for each cycle as well as for all four cycles combined. 8/1615 The appropriate sample weight was applied to combine different cycles according to the analysis guidelines of the NHANES. Binary logistic regression analysis was conducted to explore the factors that may be associated with the use of lipid screening. Both unadjusted and adjusted odds ratios (ORs) with 95% confidence intervals (CIs) were reported for each covariate. In the multivariable regression analysis, all the covariates mentioned above were included in the model. The potential multicollinearity among TC, HDL-C, and non-HDL-C was examined by variance inflation factors, which were 1.10, 1.91, and 1.99, respectively, far below the widely adopted rule-of-thumb cut-off of 10. Thus, it was considered appropriate to include the three lipid measures in the same model. All the statistical analyses were performed with SAS v9.4 (SAS Institute, Cary, North Carolina), with a P value <0.05 indicating statistical significance. III. Results Characteristics of the study population A total of 44,960 people participated in the four NHANES cycles between 2013 and March 2020. Among them, 3,600 participants aged 16– to 21 years answered questions BPQ080 and/or BPQ060 and were thus included in the estimation of the prevalence of lipid screening. The numbers of participants from the 2013–2014, 2015–2016, 2017–2018, and 2017–March 2020 cycles were 901, 753, 735, and 1211, respectively. The characteristics of the participants are presented in Table 1. The 196 weighted proportions of participants were similar across different ages and genders. 197 Approximately

half of the participants were non-Hispanic white, and more than 80% 198 of them were covered by health insurance. The weighted percentages of 199 overweight/obese, diagnosed hypertension, diagnosed diabetes, high TC, and high 200 non-HDL-C individuals were 42.44%, 3.68%, 0.79%, 6.90%, and 7.46%, 201 respectively. 202 Table 1. Characteristics of participants stratified by history of lipid screening 203 uptake. Characteristics Total (n=3600) n (weighted %) Lipid Screening Uptake Screened Never screened (n=1178) (n=2422) n (weighted %) n (weighted %) Age 16 17 18 19 20 21 Gender Male Female Ethnicity Non-Hispanic White Non-Hispanic Black Non-Hispanic Asian Mexican American Other Hispanic Other races, including multiracial Health insurance Not covered Covered BMI category Normal weight Underweight Overweight Obese Physical activity level Insufficient Sufficient Diagnosed hypertension No Yes Diagnosed diabetes 786 (19.19) 234 (15.81) 552 (20.79) 707 (16.76) 190 (14.39) 517 (17.87) 707 (16.75) 251 (17.67) 456 (16.32) 658 (14.77) 230 (15.30) 428 (14.53) 392 (17.23) 147 (19.00) 245 (16.40) 350 (15.29) 126 (17.83) 224 (14.09) 1770 (49.81) 558 (49.08) 1212 (50.15) 1830 (50.19) 620 (50.92) 1210 (49.85) 1060 (52.43) 306 (49.17) 754 (53.96) 827 (13.59) 302 (16.29) 525 (12.32) 419 (5.32) 139 (5.63) 280 (5.17) 705 (15.72) 213 (14.98) 492 (16.07) 347 (7.82) 123 (8.65) 224 (7.43) 242 (5.13) 95 (5.29) 147 (5.05) 642 (16.85) 176 (13.69) 466 (18.33) 2958 (83.15) 1002 (86.31) 1956 (81.67) 1752 (51.68) 490 (46.17) 1262 (54.25) 177 (5.89) 54 (4.73) 123 (6.42) 643 (19.63) 209 (20.60) 434 (19.17) 796 (22.81) 337 (28.50) 459 (20.15) 1875 (47.45) 551 (41.70) 1324 (50.16) 1713 (52.55) 623 (58.30) 1090 (49.84) 3467 (96.32) 1121 (95.45) 2346 (96.73) 133 (3.68) 57 (4.55) 76 (3.27) No 3571 (99.21) 1166 (98.64) 2405 (99.49) Yes 29 (0.79) 12 (1.36) 17 (0.51) HDL-C level Normal 2540 (73.83) 809 (74.37) 1731 (73.58) Low 896 (26.17) 308 (25.63) 588 (26.42) TC level Normal 3168 (93.10) 1012 (91.49) 2156 (93.86) High 268 (6.90) 105 (8.51) 163 (6.14) Non-HDL-C level Normal 3126 (92.54) 989 (90.54) 2137 (93.47) High 310 (7.46) 128 (9.46) 182 (6.53) Abbreviations: BMI, body mass index; HDL-C, high-density lipoprotein cholesterol; TC, total cholesterol. Prevalence of lipid screening uptake Among the 3,600 eligible participants, 1,178 reported a history of lipid screening when they participated in the survey, and the weighted prevalence was 32.02%. The weighted prevalences were 30.79% for 2013–2014, 33.10% for 2015–2016, 30.65% for 2017–2018, and 33.74% for 2017–March 2020 (P for trend: 0.97). Among the 1,178 participants with a history of lipid screening, 449 (weighted prevalence 11.53%) were screened “less than 1 year ago”, 353 (9.27%) were screened “1 year but less than 2 years ago”, 287 (8.03%) were screened “2 years but less than 5 years ago”, and 71 (2.70%) were screened “5 years or more ago”; the other 18 (0.49%) did not recall this information. Associations between lipid screening uptake and covariates The univariable analysis revealed that all covariates were associated with the use of lipid screening (Table 2). However, multivariable regression analysis, which included 3,282 participants with complete data on all covariates, revealed that only ethnicity, health insurance status, BMI category, and physical activity level were significantly associated with the use of lipid screening. Specifically, non-Hispanic black people (adjusted OR: 1.43; P=0.007; non-Hispanic white people as the reference), people with health insurance (adjusted OR: 1.54; P<0.001; people without health insurance as the reference), obese people (adjusted OR: 1.65; P<0.001; normal weight people as the reference) and people with sufficient physical activity (adjusted OR: 1.36; P=0.01; people with insufficient physical activity as the reference) were more likely to have a history of lipid screening uptake. Table 2. Associations between covariates and the use of lipid screening. Variable Univariable Model Multivariable Model OR (95% CI) P OR (95% CI) P Age ≤19 1.00 (ref.) 1.00 (ref.) >19 1.329 (1.327, 1.332) <0.001 1.266 (0.959, 1.670) 0.09 Gender Male 1.00 (ref.) 1.00 (ref.) Female 1.044 (1.042, 1.045) <0.001 1.036 (0.802, 1.337) 0.78 Ethnicity Non-Hispanic White 1.00 (ref.) 1.00 (ref.) Non-Hispanic Black 1.451 (1.448, 1.455) <0.001 1.432 (1.106, 1.855) 0.007 Mexican American 1.022 (1.020,

1.025) <0.001 0.982 (0.732, 1.317) 0.90 Other races* 1.216 (1.213, 1.219) <0.001 1.227 (0.932, 1.615) 0.14 Health insurance Not covered 1.00 (ref.) 1.00 (ref.) Covered 1.416 (1.412, 1.419) <0.001 1.541 (1.213, 1.958) <0.001 BMI category Normal weight 1.00 (ref.) 1.00 (ref.) Underweight 0.866 (0.862, 0.869) <0.001 0.904 (0.622, 1.313) 0.59 Overweight 1.263 (1.260, 1.265) <0.001 1.250 (0.941, 1.660) 0.12 Obese 1.662 (1.658, 1.665) <0.001 1.653 (1.276, 2.140) <0.001 Physical activity level Insufficient 1.00 (ref.) 1.00 (ref.) Sufficient 1.407 (1.405, 1.410) <0.001 1.362 (1.071, 1.732) 0.01 Diagnosed hypertension No 1.00 (ref.) 1.00 (ref.) Yes 1.409 (1.403, 1.415) <0.001 1.122 (0.692, 1.818) 0.64 Diagnosed diabetes No 1.00 (ref.) 1.00 (ref.) Yes 2.677 (2.653, 2.701) <0.001 2.768 (0.530, 14.444) 0.22 HDL-C level Normal 1.00 (ref.) 1.00 (ref.) Low 0.960 (0.958, 0.962) <0.001 0.871 (0.668, 1.135) 0.30 TC level Normal 1.00 (ref.) 1.00 (ref.) High 1.421 (1.416, 1.426) <0.001 1.034 (0.605, 1.766) 0.90 Non-HDL-C level Normal 1.00 (ref.) 1.00 (ref.) High 1.497 (1.493, 1.502) <0.001 1.314 (0.849, 2.034) 0.22 228 Abbreviations: OR, odds ratio; CI, confidence interval; BMI, body mass index; HDL-C, high-density lipoprotein 229 cholesterol; TC, total cholesterol; ref., reference group. 230 *: “Other races” include non-Hispanic Asian, other Hispanic except Mexican American, and other races, including 231 multiracial. 232 233 IV. Discussion 234 235 This study evaluated the nationwide

**prevalence of lipid screening** in young people **aged** 16– **to 21 years in**	1

the US. Nearly one-third of this population had ever received lipid screening, and 28.83% had received lipid screening in the past 5 years. Non- Hispanic black ethnicity, having health insurance, obesity (BMI≥30), and sufficient physical activity were associated with a greater chance of ever receiving

**lipid screening. The** prevalence **of lipid screening**	1

in this study was higher than those reported by most previous studies (2–27.2%) [11-17], which might be explained by several factors. First, this study was based on a nationally representative sample, whereas previous studies mostly used electronic health records (EHRs) or insurance claims data from one or several states in the US. [11-18]. Furthermore, some studies analysed well- child visit records only [12, 14, 16, 18], which may have led to underestimation of the prevalence. Second, the present study focused on young people aged 16– to 21 years, whereas previous studies focused mostly on younger populations, especially 9- to 11- year-old children [11, 14-16]. As some health problems (e.g., obesity) that may prompt the use of lipid screening are more prevalent in older adolescents [19], it is not surprising that

**the prevalence of lipid screening** was higher **in**	1

the present study. This finding was consistent with those of previous studies, which showed that older age is associated with a greater likelihood of receiving lipid screening [3, 11-13, 29]. The third possible reason was that in the present study, “lipid screening” included any such screening ever received by participants, whereas previous

studies mostly counted the screening received within particular age ranges (e.g., 9–11 years) only. The two have slightly different implications. Our finding that non-Hispanic black people were more likely to have their blood 14/1615 lipids checked is consistent with the findings of previous studies [3, 14, 15]. One possible explanation for this finding is that the obesity rate is significantly higher in non-Hispanic black youth [19]. As it is well known to the general public that obesity increases the risk of high blood cholesterol, those with a higher incidence of obesity (i.e., non-Hispanic black people) are more likely to have their blood lipids checked, as evidenced by our analysis (Table 2). The findings concerning health insurance status and BMI category are consistent with those of most previous studies [3, 11-13, 29]. It is easy to understand that people covered with health insurance are more likely to receive lipid screening and other health checkups because the insurance can cover some of or all the bills. Given that people with sufficient physical activity are more likely to receive lipid screening, these people may be more health conscious and thus more willing to take any potentially beneficial actions to improve or maintain their health. Strengths and limitations The strength of this study is that it was based on the NHANES database, which ensures the national representativeness of the sample and, consequently, the generalizability of the results. Nevertheless, the findings of this study should be interpreted with the following issues borne in mind. First, recall bias may exist. For example, the general public may not be able to recall the exact tests or procedures they underwent during a health checkup or hospital visit. This may have led to the underestimation of the prevalence of lipid screening. Second, although attempts have been made to explore the factors potentially associated with the uptake of lipid 15/1615 screening, data on family history of high blood cholesterol, education level, smoking status, and drinking status were not available for most or all the participants in this study, which precluded us from examining their influence on the outcome. V. Conclusions In conclusion, this study provides an estimate of the national prevalence of lipid screening uptake in young people aged 16– to 21 years in the US. Several factors associated with screening uptake were identified. These findings could help people understand the current situation of lipid screening in young people in the US and may serve as basic parameters in the assessment of the utilization and cost-effectiveness of related health care services. 1 2 3 4 5 6 7 8 9 10 11 12 13 14 15 16 17 18 19 20 21 22 23 24 25 26 27 28 29 30 31 32 33 34 35 36 37 38 39 40 41 42 43 44 45 46 47 48 49 50 51 52 53 54 55 56 57 58 59 60 61 62 63 64 65 66 67 68 69 70 71 72 73 74 75 76 77 78 79 80 81 82 83 84 85 86 87 88 89 90 91 92 93 94 95 96 97 98 99 100 101 102 103 104 105 106 107 108 109 110 111 112 113 114 115 116 117 118 119 120 121 122 123 124 125 126 127 128 129 130 131 132 133 134 135 136 137 138 139 140 141 142 143 144 145 146 147 148 149 150 151 152 153 154 155 156 157 158 159 160 161 162 163 164 165 166 167 168 169 170 171 172 173 174 175 176 177 178 179 180 181 182 183 184 185 186 187 188 189 190 191 192 193 194 195 204 205 206 207 208 209 210 211 212 213 214 215 216 217 218 219 220 221 222 223 224 225 226 227 236 237 238 239 240 241 242 243 244 245 246 247 248 249 250 251 252 253 254 255 256 257 258 259 260 261 262 263 264 265 266 267 268 269 270 271 272 273 274 275 276 277 278 279 280 281 282 283 284 285 286 287 288 289 290 1/1615 2/1615 4/1615 9/1615 10/1615 11/1615 12/1615 13/1615 16/1615

sources:

1 77 words / 2% - Crossref
Angela M. Thompson-Paul, Emily M. Kraus, Renee M. Porter, Samantha L. Pierce et al. "Pediatric Lipid Screening Prevalence Using Nationwide Electronic Medical Records", JAMA Network Open, 2024
